# The DUB family in *Populus*: identification, characterization, evolution and expression patterns

**DOI:** 10.1186/s12864-021-07844-3

**Published:** 2021-07-15

**Authors:** Wenqing Zheng, Liang Du

**Affiliations:** 1 Beijing Advanced Innovation Center for Tree Breeding by Molecular Design, Beijing Forestry University, Beijing, 10083 China; 2 College of Biological Sciences and Technology, Beijing Forestry University, Beijing, 100083 China

**Keywords:** *Populus*, Deubiquitinases, Evolution, Expression

## Abstract

**Background:**

The deubiquitinase (DUB) family constitutes a group of proteases that regulate the stability or reverse the ubiquitination of many proteins in the cell. These enzymes participate in cell-cycle regulation, cell division and differentiation, diverse physiological activities such as DNA damage repair, growth and development, and response to stress. However, limited information is available on this family of genes in woody plants*.*

**Results:**

In the present study, 88 DUB family genes were identified in the woody model plant *Populus trichocarpa,* comprising 44 *PtrUBP*, 3 *PtrUCH*, 23 *PtrOTU*, 4 *PtrMJD*, and 14 *PtrJAMM* genes with similar domains. According to phylogenetic analysis, the *PtrUBP* genes were classified into 16 groups, the *PtrUCH* genes into two, the *PtrOTU* genes into eight, the *PtrMJD* genes into two, and the *PtrJAMM* genes into seven. Members of same subfamily had similar gene structure and motif distribution characteristics. Synteny analysis of the DUB family genes from *P. thrchocarpa* and four other plant species provided insight into the evolutionary traits of DUB genes. Expression profiles derived from previously published transcriptome data revealed distinct expression patterns of DUB genes in various tissues. On the basis of the results of analysis of promoter *cis*-regulatory elements, we selected 16 representative *PtrUBP* genes to treatment with abscisic acid, methyl jasmonate, or salicylic acid applied as a foliar spray. The majority of *PtrUBP* genes were upregulated in response to the phytohormone treatments, which implied that the genes play potential roles in abiotic stress response in *Populus*.

**Conclusions:**

The results of this study broaden our understanding of the DUB family in plants. Analysis of the gene structure, conserved elements, and expression patterns of the DUB family provides a solid foundation for exploration of their specific functions in *Populus* and to elucidate the potential role of *PtrUBP* gene in abiotic stress response.

**Supplementary Information:**

The online version contains supplementary material available at 10.1186/s12864-021-07844-3.

## Background

Ubiquitin-mediated post-translational modification of proteins is an important means of protein function regulation [1, 2]. The dynamic balance of ubiquitination and deubiquitination is crucial for fine regulation of protein levels. Under ubiquitination, the ubiquitin molecule (Ub) binds to substrate protein by means of an isopeptic link between the glycine residue at the C-terminus and lysine residues in the substrate protein in the form of monomer or polymer chains. Three predominant enzymes mediate this process: ubiquitin-activating enzyme (E1), ubiquitin-conjugating enzyme (E2) and ubiquitin-ligase (E3) [3–7]. In contrast, deubiquitination involves the removal of ubiquitin from the ubiquitinated substrate protein, which is mediated by deubiquitinating enzymes (DUBs) [8, 9]. By removing the ubiquitin carried by the substrate protein, DUBs modulate the activity or stability of the substrate protein and recycle the ubiquitin molecules into the ubiquitin-pool [10]. The DUBs provide reversibility to the process of ubiquitination and allow modification, editing, or nixing decisions to be made by the ubiquitination machinery [11].

As in eukaryotes, plant deubiquitination enzymes can be divided into five subfamilies according to their catalytic domains [10, 12]. Four of the subfamilies are cysteine proteases, including the UBPs/USPs (ubiquitin-specific proteases), UCHs (ubiquitin C-terminal hydrolases), OTUs (ovarian tumor proteases), and MJDs (the Machado–Joseph domain). The remaining JAMM (JAB1/MPN/MOV34 proteases) subfamily comprises metalloproteinases, which are deubiquitinases whose activity depends on the metal ion zinc [8]. In additional to these five subfamilies, two novel putative DUB subfamilies comprising monocyte chemotactic protein-induced proteins (MCPIPs) and the motif interacting with Ub-containing novel DUB family (MINDY) have been identified [13–15].

To date, 53 DUBs have been identified in the model plant *Arabidopsis thaliana*. Among these DUBs are 27 UBP (UBP1–UBP27) enzymes [16], 3 UCH (UCH1–UCH3) enzymes [17], 12 OTU (OTU1–OTU5, OTLD1, OTU7–OTU12) enzymes [18], 3 MJD (JOSL, At1g07300, and At3g54130) enzymes [19], and 8 JAMM (AMSH1–AMSH3, RPN11, CSN5A, CSN5B, BRCC36A, and BRCC36B) enzymes [19–21]. The DUBs perform multiple functions in plants, such as morphogenesis [17, 22, 23], programmed cell death [24], signal transduction [25–27], and transcriptional regulation [8].

Poplar is a model plant in woody plant research owning to its rapid growth and ease of genetic transformation. Completion of the *Populus trichocarpa* genome sequence has provided convenient tool for the identification of genes and the study of gene function. In the present work, we identified 88 poplar DUB family genes, comprising 44 *PtrUBP*, 3 *PtrUCH*, 23 *PtrOTU*, 4 *PtrMJD*, and 14 *PtrJAMM* genes, using *Arabidopsis* DUBs and related conserved domains as a reference. Phylogenetic analysis showed that poplar DUB proteins were classified into different groups that varied in number of members. Further analysis was conducted to investigate gene structure, motif components, chromosome distribution, synteny analysis, expression profiles, promoter *cis*-acting elements, and response to phytohormone treatments. The analysis of gene structure and conserved motifs revealed that members of a subfamily showed a similar gene structure and motif distribution. Synteny analysis indicated that plant DUBs had undergone negative selection during evolution. Promoter analysis and phytohormone treatment indicated that PtrUBP proteins may participate in stress or phytohormone response. The present results provide valuable insights into function of the DUB family in *Populus*.

## Results

### Identification of *Populus* DUB gene family

To conduct genome-wide identification of genes in the *DUB* family of *Populus trichocarpa, Arabidopsis* UBP protein sequences were used as query sequences against the *P. trichocarpa* genomic database. A total of 88 poplar DUB members were obtained by comparison with the DUB amino acid sequences of *Arabidopsis thaliana.* The conserved domains of these sequences were analyzed using the PFam database. Ultimately, 88 DUB family genes were identified in *P. trichocarpa* genome, comprising 44 *PtrUBP*, 3 *PtrUCH*, 23 *PtrOTU*, 4 *PtrMJD*s, and 14 *PtrJAMM* genes (Table 1).

**Table 1:** Genes identified in the *Poplar trichocarpa* DUB family

Subfamily	Gene name	Gene ID	Subfamily	Gene name	Gene ID
UBP	PtrUBP1	Potri.017G055600	UBP	PtrUBP2	Potri.001G315600
UBP	PtrUBP3	Potri.007G093600	UBP	PtrUBP4	Potri.005G074900
UBP	PtrUBP5.1	Potri.016G031900	UBP	PtrUBP5.2	Potri.006G033800
UBP	PtrUBP6	Potri.003G033700	UBP	PtrUBP7	Potri.001G197400
UBP	PtrUBP8.1	Potri.016G067400	UBP	PtrUBP8.2	Potri.006G201100
UBP	PtrUBP8.3	Potri.001G214800	UBP	PtrUBP9	Potri.001G449000
UBP	PtrUBP10	Potri.011G152500	UBP	PtrUBP12.1	Potri.006G198300
UBP	PtrUBP12.2	Potri.016G064100	UBP	PtrUBP13.1	Potri.008G012600
UBP	PtrUBP13.2	Potri.010G245100	UBP	PtrUBP14.1	Potri.011G125800
UBP	PtrUBP14.2	Potri.001G408800	UBP	PtrUBP15.1	Potri.001G378900
UBP	PtrUBP15.2	Potri.011G095200	UBP	PtrUBP16	Potri.002G104800
UBP	PtrUBP17	Potri.005G156900	UBP	PtrUBP18.1	Potri.018G009500
UBP	PtrUBP18.2	Potri.018G011200	UBP	PtrUBP19	Potri.006G270600
UBP	PtrUBP20	Potri.003G092400	UBP	PtrUBP21	Potri.001G142000
UBP	PtrUBP22.1	Potri.018G123200	UBP	PtrUBP22.2	Potri.006G266100
UBP	PtrUBP22.3	Potri.018G017000	UBP	PtrUBP23.1	Potri.006G185200
UBP	PtrUBP23.2	Potri.018G107500	UBP	PtrUBP24	Potri.003G202800
UBP	PtrUBP25.1	Potri.011G112800	UBP	PtrUBP25.2	Potri.001G394600
UBP	PtrUBP26.1	Potri.005G179500	UBP	PtrUBP26.2	Potri.002G081600
UBP	PtrUBP27	Potri.007G077100	UBP	PtrUBP28	Potri.006G010100
UBP	PtrUBP29	Potri.016G014000	UBP	PtrUBP30	Potri.015G066500
UBP	PtrUBP31	Potri.012G071900	UBP	PtrUBP32	Potri.017G089250
UCH	PtrUCH1	Potri.004G130400	UCH	PtrUCH2	Potri.017G075200
UCH	PtrUCH3	Potri.003G081000	OTU	PtrOTU1.1	Potri.003G162600
OTU	PtrOTU1.2	Potri.001G067400	OTU	PtrOTU2.1	Potri.011G139500
OTU	PtrOTU2.2	Potri.001G430200	OTU	PtrOTU2.3	Potri.001G435500
OTU	PtrOTU3	Potri.016G110400	OTU	PtrOTU4.1	Potri.016G050900
OTU	PtrOTU4.2	Potri.006G057400	OTU	PtrOTU4.3	Potri.008G177400
OTU	PtrOTU4.4	Potri.010G234300	OTU	PtrOTU4.5	Potri.008G026100
OTU	PtrOTU5	Potri.014G134100	OTU	PtrOTLD1.1	Potri.009G160100
OTU	PtrOTLD1.2	Potri.004G196800	OTU	PtrOTU7	Potri.005G140500
OTU	PtrOTU9.1	Potri.008G036900	OTU	PtrOTU9.2	Potri.008G036700
OTU	PtrOTU9.3	Potri.010G225400	OTU	PtrOTU10.1	Potri.016G094700
OTU	PtrOTU10.2	Potri.006G125900	OTU	PtrOTU11.1	Potri.016G019700
OTU	PtrOTU11.2	Potri.006G021700	OTU	PtrOTU12	Potri.014G140200
MJD	PtrMJD1	Potri.001G249400	MJD	PtrMJD2	Potri.006G095700
MJD	PtrMJD3	Potri.009G043400	MJD	PtrMJD4	Potri.016G110300
JAMM	PtrBRCC36AA	Potri.001G172800	JAMM	PtrBRCC36B	Potri.002G233500
JAMM	PtrBRCC36C	Potri.014G147100	JAMM	PtrBRCC36D	Potri.010G192000
JAMM	PtrBRCC36E	Potri.010G192200	JAMM	PtrBRCC36F	Potri.008G065300
JAMM	PtrBRCC36G	Potri.008G065200	JAMM	PtrRPN11.1	Potri.002G127900
JAMM	PtrRPN11.2	Potri.014G032900	JAMM	PtrAMSH1	Potri.015G045800
JAMM	PtrAMSH2	Potri.010G041200	JAMM	PtrAMSH3	Potri.010G141100
JAMM	PtrCSN5A	Potri.018G006100	JAMM	PtrCSN5B	Potri.006G275100

Details regarding the poplar DUB family genes, including the gene identifier (Gene ID), the number of amino acid of protein, the length of the coding sequence (CDS) region, theoretical isoelectric point (pI), molecular weight (MW), predicted subcellular location, and chromosome mapping, are listed in Additional file 1: Table S1. Among the 88 DUB proteins, PtrMJD3 was the smallest protein with 112 amino acids (aa), whereas the largest protein identified was PtrUBP32 with 2100 amino acids. The pI of the DUB proteins ranged from 4.45 (PtrMJD4) to 9.77 (PtrMJD3). The MW ranged from 12.77 (PtrMJD3) to 239.34 kDa (PtrUBP32). The majority of the proteins were predicted to be localized in the nucleus, but some of the proteins were predicted to be localized in the chloroplasts, cytosol, endoplasmic reticulum, mitochondria, or vacuole. In addition, three proteins (PtrUBP13.2, PtrUBP16, and PtrUBP14.2) were predicted to target two organelles. The chromosome mapping results showed that the DUB genes were distributed on almost every chromosome, except for chromosomes 13 and 19.

### Analysis of conserved domains, phylogenetic relationships, and classification of the poplar DUB family

**Fig. 1:**
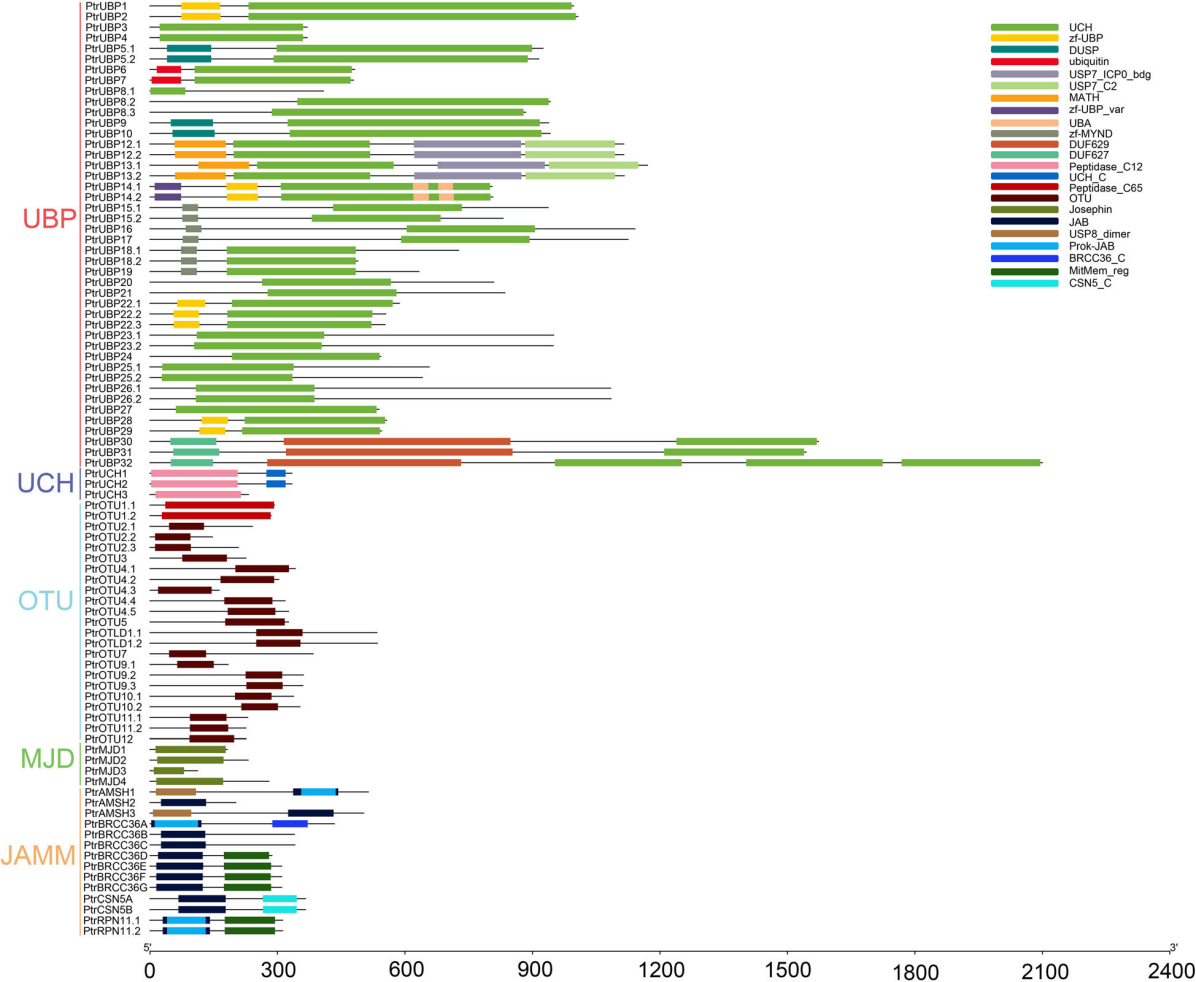
Conserved domains in the UBP, UCH, OTU, MJD, and JAMM subfamilies of the *Poplar thrichocarpa* DUB family. Different colors represent different conserved domains. The PtrUBP proteins contained the conserved UCH and certain other domains, such as zf-UBP, DUSP, ubiquitin, MATH, and DUF629. The PtrUCH proteins all contained the Peptidase_C12 domain. The PtrOTU proteins contained the conserved Peptidase_C65 or OTU domain. The PtrMJD and PtrJAMM proteins contained the conserved Josephin and JAB domains

Analysis of conserved domains of the five subfamilies of DUB enzymes was conducted using the Pfam database. The 44 PtrUBP proteins exhibited a typical conserved UCH domain with cysteine (Cys) and histidine (His) boxes [28]. In addition, the PtrUBP proteins contained other conserved non-UBP domains such as zf-UBP, DUSP, ubiquitin, and MATH (Fig. 1). The PtrUBP proteins were grouped into 16 groups in accordance with the grouping of the AtUBP proteins [29]. The Peptidase_C12 domain was included in all three PtrUCH proteins, and the UCH_C domain was detected only in PtrUCH1 and PtrUCH2 proteins. The OTU domain was present in most of the PtrOTU proteins except PtrOTU1.1 and PtrOTU1.2, both of which harbored Peptidase_C65. For PtrMJD and PtrJAMM proteins, the typical motifs were the Josephin domain and JAB domain, respectively. Additional domains were present in the PtrJAMM proteins such as Mitmem_reg and CSN5_C.

**Fig. 2:**
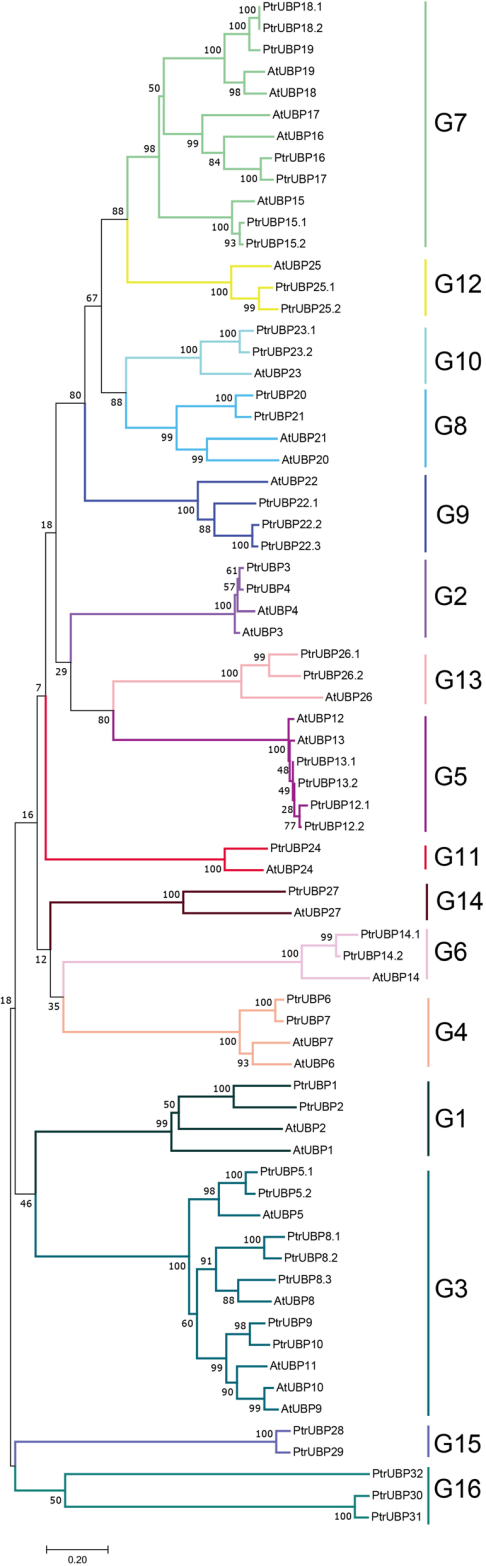
Phylogenetic relationships of UBP proteins from *Arabidopsis thaliana* and *Populus trichocarpa.* The phylogenetic tree was constructed using the Maximum Likelihood (ML) method with MEGA7.0. Different colors indicate different groups. The numbers nearby the internal nodes represent the degree of confidence. The bar represents the branch length at the bottom of the figure

To analyze the phylogenetic relationships among the DUB proteins of *Arabidopsis thaliana* and *Populus trichocarpa,* five phylogenetic trees were constructed on the basis of alignments of the full-length DUB protein sequences from *Arabidopsis thaliana* (27 UBPs, 12 OTUs, 3 MJDs, 3 UCHs, and 8 JAMMs) and *Populus trichocarpa* (44 UBPs, 23 OTUs, 4 MJDs, 3 UCHs, and 14 JAMMs). The phylogenetic reconstruction for the UBP subfamily is shown in Fig. 2 and those of the other subfamilies are shown in Additional file 7: Fig. S1. Detailed information for the *Arabidopsis* DUB family is provided in Additional file 2: Table S2. On the basis of the UBPs phylogenetic tree, all the sequences were classifiable into 16 groups. The number of members in the different groups varied. G11 and G14 each contained only one member, two members were included in G1, G2, G4, G6, G8, G10, G12, G13, and G15, three in G9 and G16, four in G5, and seven in G3 and G7. The PtrOTUs were divided into eight groups. One PtrOTU member was included in G3, G5 and G7, two in G1 and G6, three in G2, five in G4, and eight in G8. The PtrUCH subfamily was mainly classified into two groups. PtrMJD proteins were resolved into two groups, G1 and G2. PtrJAMM proteins were grouped into seven groups.

### Gene structure and motif distribution of the poplar DUB family

**Fig. 3:**
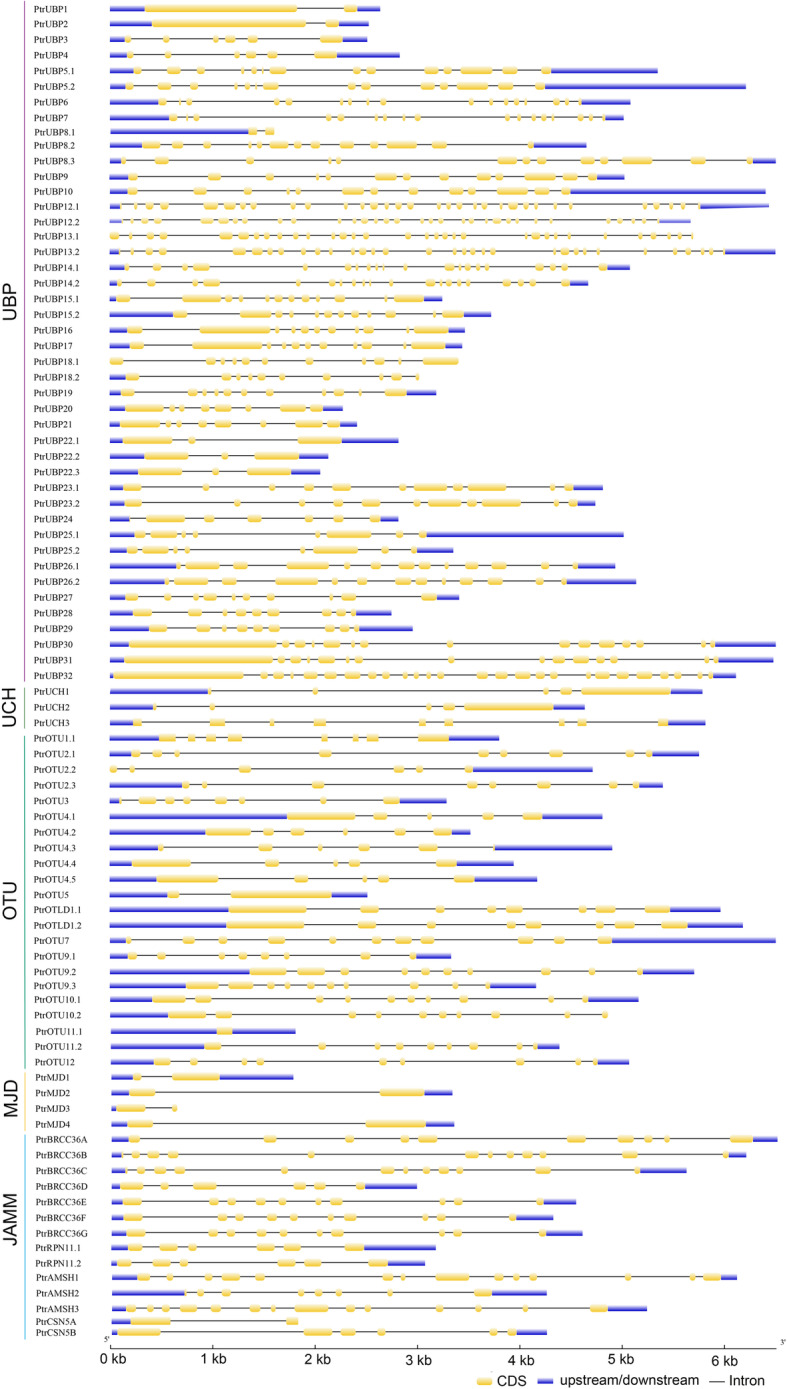
Exon–intron distribution of *Poplar trichocarpa* DUB genes. The exon–intron distributions were sorted according to the UBP, UCH, OTU, MJD, and JAMM subfamilies. Coding sequence (CDS), intron, and upstream/downstream regions are indicated by a yellow rectangular box, black line, and blue rectangular box, respectively

To gain additional insight into the evolution of the DUB family in poplar, the exon–intron organization of all the identified poplar DUB family genes was examined. The gene structure differed among subfamilies, but little difference in gene structure was observed among members of the same subfamily, especially the *PtrUBP* subfamily (Fig. 3). For example, in the *UBP* subfamily, all G1 members had two exons and one intron, and all G2 genes contained six exons and five introns. The members of the G5 group of the *PtrUBP* subfamily contained the highest number of exons (32). The 5′- and 3′- untranslated regions (UTRs) were both present in most poplar DUB family genes except for *PtrUBP8.1, PtrUBP18.2, PtrOTU10.2, PtrMJD3*, and *PtrCSN5A*, of which had only 5′-UTR, *PtrUBP18.1* and *PtrUBP13.1* contained neither UTR, and *PtrOTU2.2* had only the 3′-UTR.

**Fig. 4:**
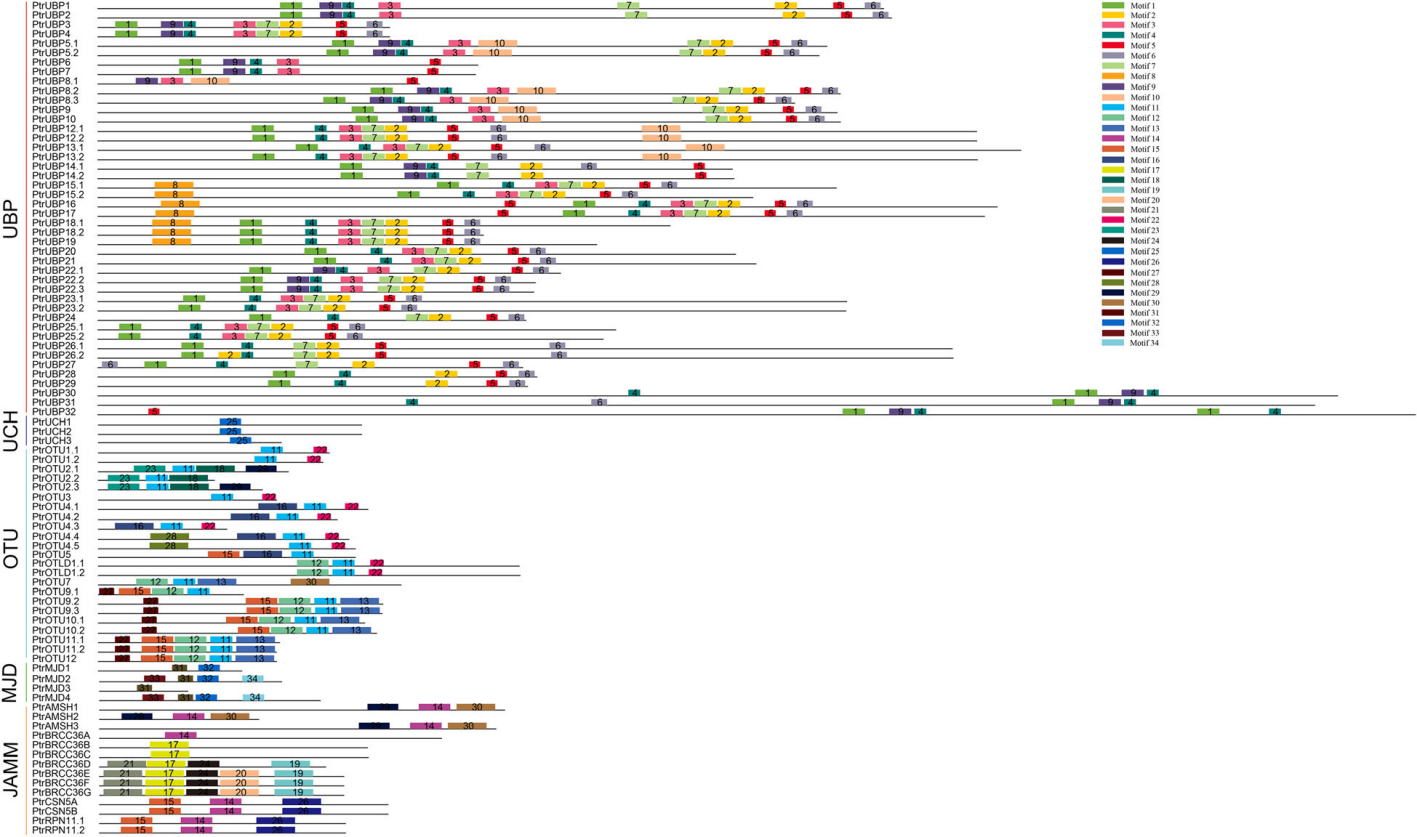
Motif distribution of *Poplar trichocarpa* DUB proteins. The same subfamily has similar motif distribution characteristics. Different motifs are indicated by boxes with different colors. The length of the black line represents the size of the protein. The PtrUBP subfamily contains motifs 1–10. The other subfamilies contain motifs 11–34

To analyze the DUB protein motif distribution, the motif characteristics of all poplar DUB proteins were examined using the MEME online tool (Fig. 4). A total of 34 motifs were identified. Detailed information on the length and sequence of the motifs were showed in Additional file 3: Table S3. The members within the same subfamily usually shared a similar motif composition. For example, motif 11 was unique to OTU subfamily, whereas motif 25 was specific to the UCH subfamily. Motif 31 was detected in all members of the MJD subfamily.

### Expression profiling of poplar DUB family genes by RNA-sequencing

**Fig. 5:**
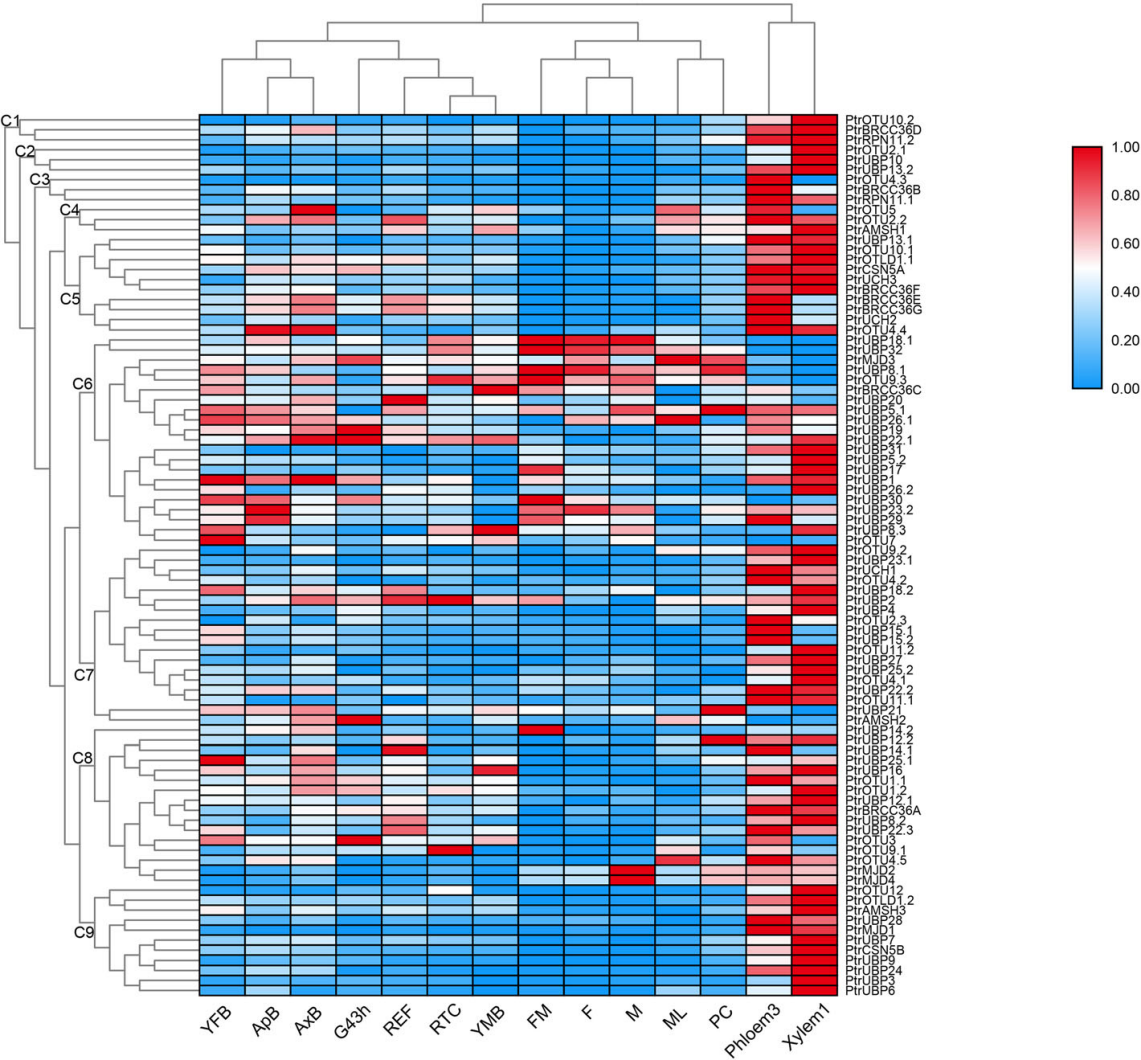
Expression profiles of the *Poplar trichocarpa* DUB family in different vegetative tissues and stages of reproductive development. FM, female catkin prior to seed release; F, female catkin post-fertilization; M, male catkin; ML, mature leaf; PC, phloem, cortex, and epidermis; G43h, germinated seedling sampled 43 h post imbibition; YFB, newly initiated female floral buds; ApB, actively growing shoot apex; AxB, axillary bud; REF, roots from field-grown trees; RTC, roots from plants in tissue culture; YMB, newly initiated male floral buds; Phloem3, developing phloem; Xylem1, developing xylem

To study the expression patterns of all 88 poplar DUB family genes, we downloaded and analyzed transcriptome data for different vegetative tissues and stages of reproductive development from a public database generated in a previous study [30]. Hierarchical clustering of the heatmap revealed clear differential expression of the DUB genes in different poplar tissues and development stages (Fig. 5). RNA-sequencing data for the 88 poplar DUB family genes were listed in Additional file 4: Table S4.The analyzed tissues or developmental stages were grouped into three clusters: one cluster consisted of the DUB expression patterns in FM (female catkin prior to seed release), F (female catkin post-fertilization), M (male catkin), ML (mature leaf), and PC (phloem, cortex, and epidermis); a second cluster was composed of G43h (germinated seedling sampled 43 h post- imbibition), YFB (newly initiated female floral buds), ApB (actively growing shoot apex), AxB (axillary bud), REF (roots from field-grown trees), RTC (roots from plants in tissue culture), and YMB (newly initiated male floral buds); and Phloem3 (developing phloem) and Xylem1 (developing xylem) formed a third cluster. On the basis of the expression profiles in the 14 tissues, the poplar DUB family genes were grouped into nine clusters (C1 to C9) (Fig. 5). The three genes grouped in C1 were highly expressed in Xylem1 and Phloem3 tissues. Genes clustered in C6 were highly expressed in YFB and ApB. The majority of the genes (except *PtrOTU9.3, PtrUBP14.2, PtrUBP17, PtrAMSH2, PtrMJD4, PtrMJD2, PtrUBP30, PtrUBP23.2,* and *PtrUBP29*) showed lower expression levels in FM, F, and M (Fig. 5). In addition, many genes (except *PtrUBP32, PtrUBP8.1, PtrOTU9.3,* and *PtrUBP18.1*) showed higher expression levels in Xylem1 tissues.

### Chromosomal distribution and synteny analysis of poplar DUB family genes

**Fig. 6:**
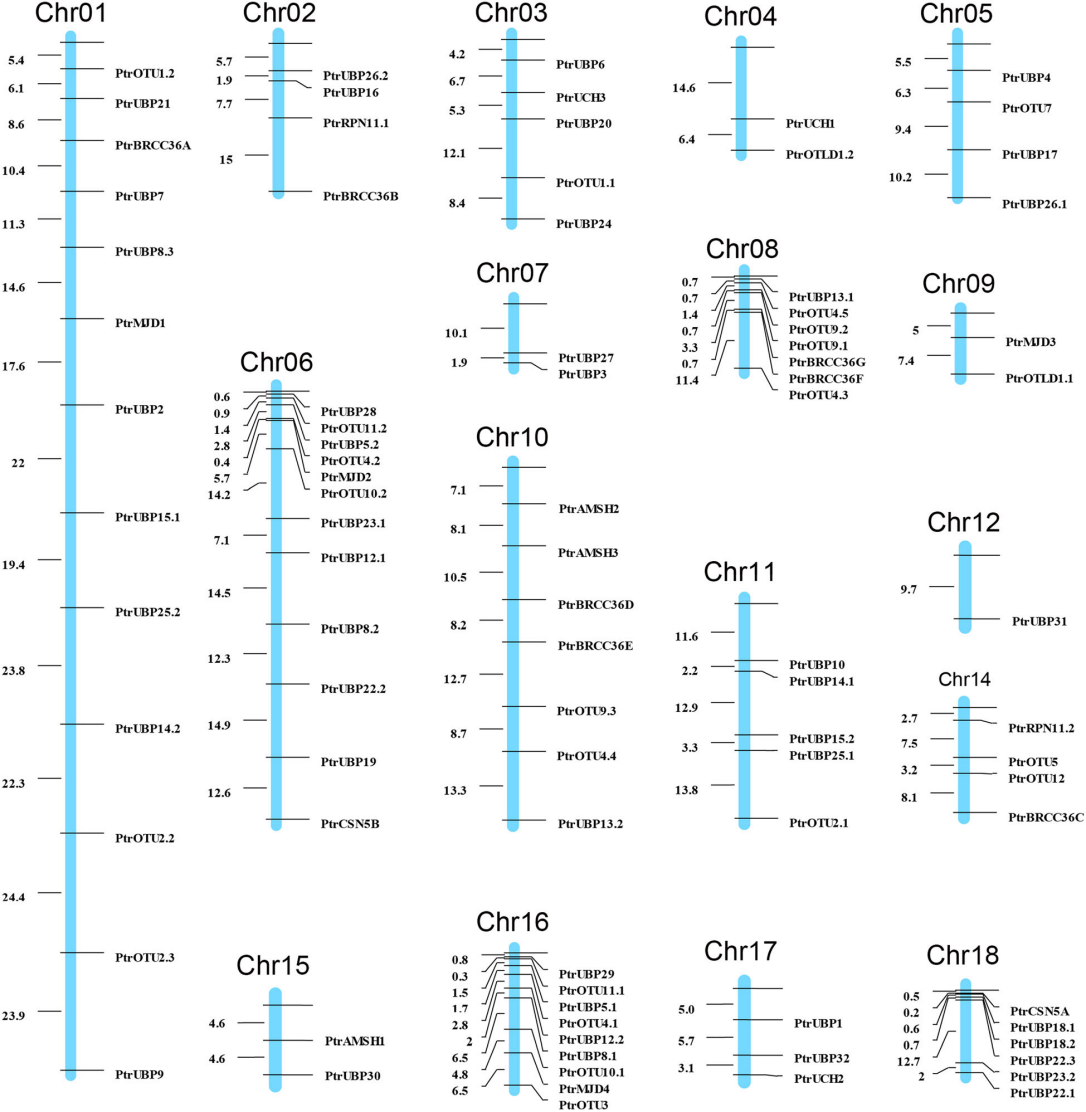
Diagrammatic illustration of the chromosomal distribution of *Poplar trichocarpa* DUB family genes. Cylinders represent different chromosomes

The poplar DUB family genes were unevenly distributed across the 19 poplar Chromosomes (Chr), except Chr13 and Chr19 (Fig. 6). Chr1 contained the highest number of *DUB* genes (13). Some chromosomes (e.g., Chr16 and Chr6) had a relatively high number of genes, whereas others contained few DUB genes, such as Chr4, Chr7, Chr9, Chr15 and Chr12. Chr12 contained only one DUB gene. In addition, 44 paralogous pairs comprising 88 DUB genes were identified (Additional file 5: Table S5).

**Fig. 7:**
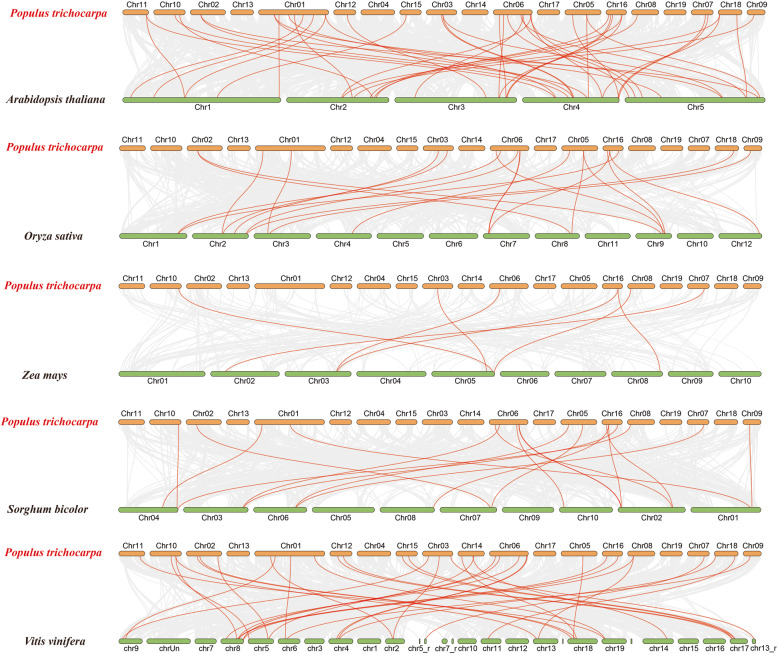
Synteny analysis of DUB family genes between poplar and five representative plant species. Gray lines indicate the collinear blocks within poplar and the other five plant species, and the red lines highlight the syntenic DUB gene pairs. The other five species from top to bottom are *Arabidopsis thaliana, Oryza sativa, Zea mays, Sorghum bicolor*, and *Vitis vinifera*

To further examine the phylogenetic relationships of the poplar DUB family, we constructed five comparative syntenic maps of *Populus trichocarpa* associated with five representative species, comprising two dicotyledons (*Arabidopsis thaliana* and *Vitis vinifera*) and three monocotyledons (*Oryza sativa, Zea mays,* and *Sorghum bicolor*) (Fig. 7). A number of poplar DUB genes showed a syntenic relationship with genes from *Arabidopsis*, *O. sativa*, *Z.mays*, *S. bicolor*, and *V. vinifera* (Additional file 5: Table S5). The numbers of orthologous pairs between the other five species (*Arabidopsis*, *O. sativa*, *Z. mays*, *S. bicolor*, and *V. vinifera*) were 62, 27, 9, 24, and 55. Some poplar DUB genes were associated with three or four syntenic gene pairs. For example, *PtrUBP12.2* was associated with four gene pairs in *Populus* and *Arabidopsis.*

To further explore the evolutionary and divergence patterns of the DUB genes, the *Ka/Ks* ratio for each DUB gene pairs was calculated. In principle, a *Ka/Ks* ratio less than 1, equal to 1 and greater than 1 represents negative selection, neutral selection, and positive selection, respectively [31, 32]. All segmental duplicated poplar DUB gene pairs and orthologous DUB gene pairs had *Ka/Ks* < 1, which indicated that the poplar DUB family genes might have experienced strong purifying selective pressure during evolution.

### Analysis of the putative promoter regions of the poplar DUB family genes

**Fig. 8:**
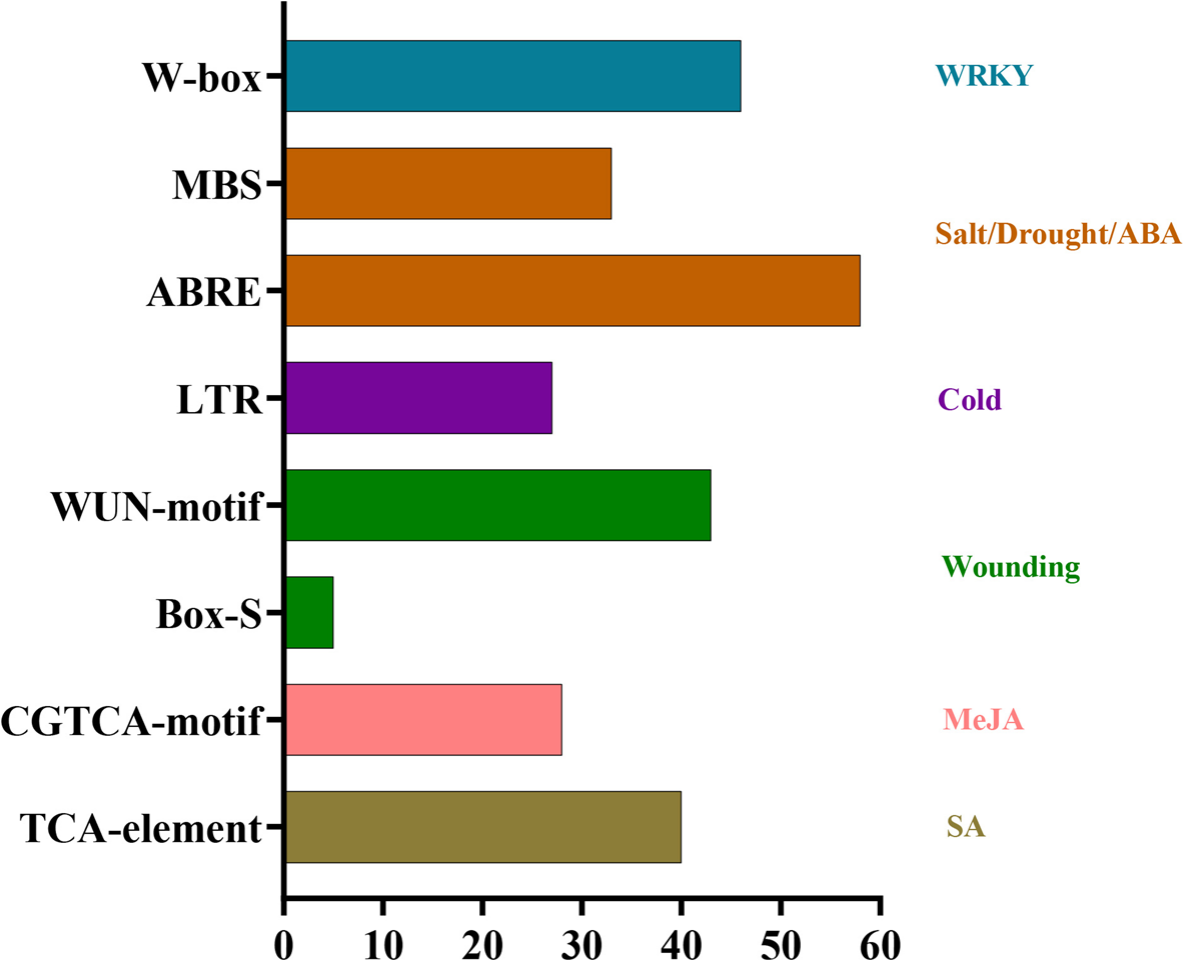
Number of genes containing various *cis*-elements in the promoter region. TCA-element, SA-responsive element; CGTCA-motif, MeJA-responsive element; Box-S and WUN-motif, wounding responsive element; LTR, low-temperature-responsive element; ABRE, ABA-responsive element; MBS, MYB binding site; W-box, WRKY binding site

To examine the regulatory elements in the promoter regions of the poplar DUB family genes, 2000 bp of the genomic sequence upstream of the start codon ATG were selected as the putative promoter region and analyzed using the Plant–CARE database. Many *cis*-acting elements associated with stress or phytohormone response were present in the promoter of the DUB genes (Fig. 8). For example, the promoter contained abundant *cis*-acting elements, such as salicylic acid (SA)- responsive elements (TCA-elements), methyl jasmonate (MeJA) responsive elements (CGTCA-motif), Box-S and WUN motifs involved in wounding stress, low-temperature responsive (LTR) element that participates in the response to cold stress, abscisic acid-responsive element (ABRE) and MYB binding site (MBS) motifs associated with response to salt, drought, and abscisic acid (ABA), and the W-box (WRKY binding site) motif. Details of the *cis*-acting elements, including specific motifs detected in each gene, are listed in Additional file 6: Table S6. TCA-elements and CGTCA-motif were detected in the promoter of 40 and 28 poplar DUB family members, respectively. Five and 43 promoters contained Box-S and WUN motifs associated with wounding stress, respectively. The LTR element was observed in 27 promoters of poplar DUB family genes. The MBS and ABRE motifs were abundant in the promoter of poplar DUB family genes. In addition, the W-box was detected in the promoter of 46 poplar DUB family genes.

### Quantitative RT-PCR analysis of poplar DUB family genes in response to phytohormone treatments

**Fig. 9:**
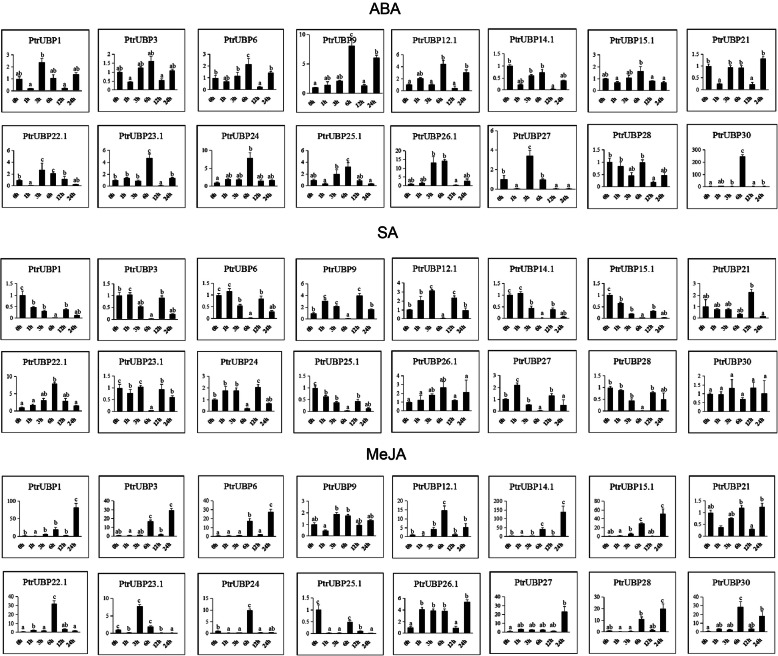
Expression profiles of 16 selected PtrUBP genes in response to various phytohormone treatments. The phytohormones used here were ABA, SA, and MeJA. *Ptr18S* gene was selected as a reference gene. The data were counted from at least three biological replicates. The *p*-values of all the data were calculated using duncan’s test, labeled with a, ab, b, or c

Among members of the DUB family, the UBP subfamily has been reported to participate in the regulation of ABA [33] and MeJA [27, 34, 35] pathway. To investigate whether *PtrUBP* genes expressions was influenced by different phytohormone treatments, the expression of 16 *PtrUBP* genes *(PtrUBP1, PtrUBP3, PtrUBP6, PtrUBP9, PtrUBP12.1, PtrUBP14.1, PtrUBP15.1, PtrUBP21, PtrUBP22.1, PtrUBP23.1, PtrUBP24, PtrUBP25.1, PtrUBP26.1, PtrUBP27, PtrUBP28,* and *PtrUBP30*) from 16 different groups were detected in response to ABA, SA, and MeJA treatment (Fig. 9). Overall, some *PtrUBP* genes were induced or suppressed by different treatments. Each gene showed a different expression trend in different treatments. For example, *PtrUBP1* was suppressed in the SA treatment but induced in the MeJA treatment. The expression level of most genes peaked at 6 h. However, the expression levels of some genes peaked at 1, 3, 12, or 24 h. For instance, the expression of *PtrUBP3, PtrUBP6, PtrUBP14.1,* and *PtrUBP27* under SA treatment peaked at 1 h.

## Discussion

Deubiquitinases are important proteolytic enzymes in ubiquitin system and participate in plant growth and development [36–38], immune stress response [26], and signal transduction pathways [39]. In previous research, 53 deubiquitinases were identified from *Arabidopsis thaliana* [19]. The deubiquitinases of *Populus trichocarpa* have not been described previously. In the current study, 88 poplar deubiquitinases were identified, consisting of 44 UBPs, 3 UCHs, 23 OTUs, 4 MJDs, and 14 JAMMs. The number of UCH subfamily members is identical to that of *Arabidopsis,* whereas the other four subfamilies contain considerably more genes than those in *Arabidopsis*. Given that the genome size of *Arabidopsis* is substantially smaller than that of poplar [40, 41], the same number of UCH members in the two species indicates that the UCH subfamily might be more conserved than the other four DUB subfamilies.

On the basis of the prediction of conserved domains, it is particularly notable that the PtrUCH3 protein does not harbor a UCH_C domain, but instead contains the Peptidase_C12 conserved domain. The PtrOTU1.1 and PtrOTU1.2 proteins do not carry the OTU domain and instead contain the Peptidase_C65 conserved domain. According to the EMBL-EBI database, Peptidase_C12 (PF01088) and Peptidase_C65 (PF10275) belong to the Ubiquitin carboxyl-terminal hydrolase family 1 and Peptidase C65 Otubain family, respectively. However, further experiments are needed to verify the subfamilial affinities of these proteins.

The predicted subcellular location of the poplar DUB family proteins was the chloroplasts, cytosol, endoplasmic reticulum, mitochondria, vacuole, or nucleus. Different locations are indicative of different functions. For example, AtUBP3 and AtUBP4 located in the nucleus are involved in pollen development [42, 43]. AtUBP11 and AtUBP12 located in the nucleus or cytoplasm and may affect the expression of many genes, hence proteins that are localized to the cytoplasm extensively influence plant growth and development, including plant immunity [44], flowering time, circadian rhythm [27], and jasmonic acid signaling [45]. The *AtUBP12* homolog in tobacco *NtUBP12* is a negative regulator of plant immunity [44]. AtUBP27 is associated with the mitochondria and is involved in mitochondrial morphogenesis [46].

### Expansion of the PtrUBP subfamily suggests functional diversification

According to the phylogenetic analysis, PtrUBP subfamily members were classified into 16 groups, which represents two more groups than AtUBPs. PtrUBP28 and PtrUBP29 belong to the novel group 15 (G15), and PtrUBP30, PtrUBP31, and PtrUBP32 belong to the novel group 16 (G16). Each of the 16 groups contained similar protein domains. Compared with *Arabidopsis*, PtrUBP members in G16 contained two specific conserved domains DUF627 and DUF629, which indicates the complexity of the PtrUBP subfamily.

The synteny analysis of the poplar DUB family showed that DUB gene pairs had *K*_*a*_*/K*_*s*_ < 1, which suggests that the poplar DUB family genes might have experienced strong purifying selective pressure during evolution. In principle, the value of *K*_*a*_*/K*_*s*_ ratio less than 1, equal to 1, and greater than 1 represents negative selection, neutral selection, and positive selection, respectively. However, the *K*_*a*_*/K*_*s*_ ratio is not the only standard measure of selection pressure. The determination of the selection pressure may require a further analysis [47].

### Expression patterns of poplar DUB family members indicate their potential roles in plant development

Analysis of DUB gene expression patterns from different tissues and developmental stages provide insight into the functions of DUB genes in the growth and development of *Populus*. We profiled DUB gene expression on the basis of published transcriptome data. A large number of DUB genes were highly expressed in the xylem, indicating that they may be involved in the development of xylem. Previous studies show that the UBP subfamily is involved in diverse processes in the plant development, including embryo development [16, 22], seed development [34, 37, 48], seed size [23], leaf cell size [49], and pollen development or transmission [42]. However, little information is available on the role of the UBP subfamily in xylem or cambium development, inferring that the functions of genes in the same subfamily differ among plant species.

### Poplar DUB family members play an important role in phytohormone-related signaling response

Plants have evolved various mechanisms to resist different stresses in response to diverse environmental conditions [50]. The DUB family has been reported to be involved in phytohormone and stress response, especially the UBP family [16, 33, 45]. Plants generate a variety of signaling molecules in response to stress such as abscisic acid (ABA), salicylic acid (SA), methyl jasmonate (MeJA), and ethylene (ET). Additionally, elements of gene promoter play important roles in growth and developmental processes of plants, especially in the determination of the tissue-specific expression or stress-responsive regulation of genes [51, 52]. Based on previous research, we conducted an analysis of *cis*-acting elements. The poplar DUB family genes contained abundant *cis*-acting elements, including TCA-elements (SA response), CGTCA-motif (MeJA response), Box-S, WUN motif (wounding stress response), LTR (cold stress response), ABRE, MBS motif (salt/drought/ABA response), and W-box (WRKY binding site) [51–56]. Indeed, plant UBPs show potential in the regulation of stress responses [33]. In view of the important role of the UBP subfamily in abiotic stress resistance, the expression level of PtrUBP genes was quantified by qRT-PCR analysis in response to ABA, SA, and MeJA treatments. *PtrUBP1* and *PtrUBP22.1* were significantly induced in response to MeJA treatment, implying that they may play an important role in the jasmonic acid signaling pathway. In *Arabidopsis*, The AtUBP1(the homolog of PtrUBP1) is involved in canavanine (CAN) resistance, indicating that PtrUBP1 maybe perform different functions in the *Populus* [29]. Therefore, additional experimentation is needed to verify the function of PtrUBP1.

## Conclusion

The DUB family is involved in various growth and developmental processes in plants. In this study, a total of 88 poplar DUB family genes comprising 44 *PtrUBP*, 3 *PtrUCH*, 23 *PtrOTU*, 4 *PtrMJD*, and 14 *PtrJAMM* genes, were identified. Analysis of conserved domains, exon–intron structure and motif distribution showed that members of the same subfamily had similar characteristics. Synteny analysis of DUB family genes from different plant species provided valuable insight into the evolutionary characteristics of poplar DUB family members. The expression patterns of poplar DUB family genes indicate that DUB genes play important roles in poplar growth and development in different tissues and at different developmental stages. The expression of poplar UBP genes in response to different phytohormone treatments offers a new perspective on the role in the stress or hormone response of poplar UBPs. The present results provide a valuable resource for future exploration of the biological functions of DUB family genes in poplar.

## Materials

### Identification of *Populus trichocarpa* DUB family members

The sequences of DUB family members of *Arabidopsis thaliana* were downloaded from The Arabidopsis Information Resource (TAIR) database (https://www.arabidopsis.org/). The accession numbers are listed in Additional file 2: Table S2. To identify the *Populus* DUB family members, we used the amino acid sequence of the *Arabidopsis* DUB family as the query sequences for a Blast search of the *Populus trichocarpa* genome database (version 3.0; https://genome.jgi.doe.gov/portal/Poptr1/Poptr1.home.html)*.* Th poplar DUB family members were further confirmed by analysis of the conserved domains using the EMBL-EBI Pfam database (http://pfam.xfam.org/). The number of amino acids, length of the coding sequence (CDS), the theoretical isoelectric point (pI), and molecular weight (MW) of amino acids were predicted using the ExPASy-Prot Param online tool (https://web.expasy.org/protparam/) [57]. The subcellular location of *Populus* DUB proteins was predicted using the WoLF PSORT: Advanced Protein Subcellular Localization Prediction Tool (https://www.genscript.com/wolf-psort.html?src=leftbar) [58].

### Determination of conserved domain and phylogenetic analysis

The conserved domains of all identified poplar DUB family genes were determined using the EMBL-EBI Pfam32.0 batch sequence search tool (http://pfam.xfam.org/search#tabview=tab1) and confirmed by searching the Conserved Domain Database (CDD) of the National Center for Biotechnology Information (NCBI; https://www.ncbi.nlm.nih.gov/Structure/cdd/wrpsb.cgi) [59]. The protein sequences of *Arabidopsis* DUB family members were obtained from the TAIR database on the basis of the gene ID [19, 60, 61]. The protein sequences of rice DUB family members were downloaded from the Rice Genome Annotation Project (http://rice.plantbiology.msu.edu/) [62]. The protein sequences of *P. trichocarpa* DUB family members were downloaded from the Phytozome12.1 database (https://phytozome.jgi.doe.gov). The protein sequences were aligned using Muscle. Phylogenetic trees were constructed with MEGA 7.0.26 using the maximum likelihood method with 1000 bootstrap replications.

### Gene structure and motif analysis

Firstly, the CDS and nucleotide sequences of *P. trichocarpa* DUB family genes were downloaded from the Phytozome 12.1 database (https://phytozome.jgi.doe.gov). The gene structure was determined using these sequences with the Gene Structure Display Server (GSDS) (http://gsds.cbi.pku.edu.cn) [63]. The conserved motifs in the *P. trichocarpa* DUB proteins were identified by using the MEME Suite (version 5.3.3; http://meme-suite.org) with the following criteria: maximum number of different motifs 10 for PtrUBP, 24 for other subfamilies; motif sites to be distributed in sequences as zero or one occurrence per sequence (zoops); and using the classic motif discovery mode [64].

### Chromosomal distribution and synteny analysis

The *P. trichocarpa* DUB family genes were mapped to the corresponding position of *Populus* chromosomes on the basis of the genome database and visualized using Circos [65]. Gene duplication events were analyzed using the Multiple Collinearity Scan toolkit (MCScanX) [66]. The syntenic analysis maps were drawn using the Dual Systeny Plotter software (https://github.com/CJ-Chen/TBtools) [67]. The synonymous substitution rate (*Ks*) and nonsynonymous substitution rate (*Ka*) were computed using KaKs_Calculator 2.0 [68].

### Putative promoter region analysis

The 2000-bp upstream genomic sequences of the *P. trichocarpa* DUB family genes were selected as the putative promoter regions. The inclusive *cis*-regulatory elements were identified and analyzed using the Plant CARE database (http://bioinformatics.psb.ugent.be/webtools/plantcare/html/) [69].

### Plant materials treatment, RNA extraction and gene expression analysis

Poplar 84 K (*Populus alba*×*Populus glandulosa)*, an aspen hybrid poplar from Korea, was used in the study. Poplar trees were grown in a greenhouse maintained at 25°Cunder a 16-h/ 8-h (light/dark) photoperiod. Poplar seedlings grown in greenhouses for about three months were treated with phytohormones (ABA, SA, and MeJA). The seedlings were sprayed with Murashige and Skoog (MS) liquid medium supplemented with 100 μM ABA, SA, or MeJA, and sampled at 1, 3, 6, 12, and 24 h after treatment. Seedlings sprayed with MS medium solution were treated as the control and sampled at 0 h. All collected tissue samples were immediately frozen in liquid nitrogen and stored at-80 °C for subsequent analysis.

Total RNA of treated samples was extracted using the RNAprep Pure Plant Kit (TIANGEN - DP419, Beijing, China). Integrity and concentration of RNA were detected using agarose gel electrophoresis and Nanodrop micro-spectrophotometer respectively. The cDNA was synthesized using the TransScript® One-Step gDNA Removal and cDNA Synthesis SuperMix Kit (TransGen - AT311, Beijing, China) in accordance with the manufacturer’s instruction. Quantitative RT-PCR analysis was conducted on the Bio-Rad Real-time instrument using the TransStart® Top Green qPCR SuperMix (TransGen-AQ132, Beijing, China). The 18S rRNA gene was used as an internal control. Primers were designed with the NCBI Primer-BLAST tool (http://www.ncbi.nlm.nih.gov/tools/primer-blast/) on the basis of the CDS sequences. The PCR reaction was conducted as follows: denaturation at 94°Cfor 30 s, then 40 cycles of denaturation at 94 °C for5 s, annealing at 60 °C for30 s, and extension at 72 °C for30 s. The quantitative RT-PCR data were analyzed using the 2^−△△Ct^ method. The gene expression heatmaps for different tissues were generated using the TBtools software [67].

## Supplementary Information

**Additional file 1 Table S1.** Detailed information on the *Populus* DUB family.

**Additional file 2 Table S2.** Detailed information of on the *Arabidopsis* DUB family.

**Additional file 3 Table S3.** Detailed information on the MEME motif sequences about DUB proteins in *Populus trichocarpa*.

**Additional file 4 Table S4.** RNA-sequencing data for 88 PtrDUB genes used in this study.

**Additional file 5 Table S5**. The value of *Ka, Ks,* and *Ka/Ks* for syntenic gene pairs from different species.

**Additional file 6 Table S6** Detailed information of *cis*-acting elements of 88 PtrDUB genes.

**Additional file 7 Fig. S1** Evolutionary relationships among *Poplus trichocarpa* and *Arabidopsis thaliana* DUB subfamily members.

## Data Availability

All data supporting the conclusions of this article are included in the article and its additional files.

## Abbreviations

DUB: Deubiquitinase
UBP: Ubiquitin-specific protease
UCH: Ubiquitin C-terminal hydrolase
OUT: Ovarian tumor protease
MJD: The Machado–Joseph domain
JAMM: JAB1/MPN/MOV34 protease
MCPIP: Monocyte chemotactic protein-induced protein
MINDY: Motif interacting with Ub-containing novel DUB family
Gene ID: Gene identifier
pI: Theoretical isoelectric point of protein
MW: Molecule weight
CDS: The coding sequence
CSN: COP9 Complex subunit
UTR: Untranslated region
SA: Salicylic acid
MeJA: Methyl jasmonate
LTR: Low-temperature responsive
ABRE: Abscisic acid-responsive element
MBS: MYB binding site
ABA: Abscisic acid
W-box: WRKY binding site
WUN-motif: Wound-responsive element
qRT-PCR: Quantitative real-time PCR
*Ptr*: *Populus trichocarpa*
Chr: Chromosome
