# Supplementary material for: The DUB family in Populus: identification, characterization, evolution and expression patterns
Source: BMC Genomics. 2021 Jul 15;22:541. doi: 10.1186/s12864-021-07844-3 (PMC8281628; doi:10.1186/s12864-021-07844-3)
Supplement: Supplementary file 1 — Additional file 1 Table S1. Detailed information on the Populus DUB family. [file 12864_2021_7844_MOESM1_ESM.docx]

Table S1. Detailed information on the *Populus* DUB family.

| **subfamily** | **Gene name** | **Gene ID** | **CDS length(bp)** | **Amid acid length(aa)** | **pI** | **MW(Da)** | **Predicted subcellular location** | **Chromosome No.** |
| --- | --- | --- | --- | --- | --- | --- | --- | --- |
| UBP | PtrUBP1 | Potri.017G055600 | 2994 | 997 | 5.22 | 109246.8 | nucleus | 17 |
| UBP | PtrUBP2 | Potri.001G315600 | 3024 | 1007 | 5.27 | 109986.6 | nucleus | 1 |
| UBP | PtrUBP3 | Potri.007G093600 | 1113 | 370 | 5.79 | 42106.52 | nucleus | 7 |
| UBP | PtrUBP4 | Potri.005G074900 | 1113 | 370 | 5.84 | 42066.55 | nucleus | 5 |
| UBP | PtrUBP5.1 | Potri.016G031900 | 2778 | 925 | 5.71 | 104347.9 | nucleus | 16 |
| UBP | PtrUBP5.2 | Potri.006G033800 | 2748 | 915 | 5.90 | 103662.9 | nucleus | 6 |
| UBP | PtrUBP6 | Potri.003G033700 | 1449 | 482 | 5.79 | 54210.48 | nucleus | 3 |
| UBP | PtrUBP7 | Potri.001G197400 | 1440 | 479 | 6.34 | 54282.93 | nucleus | 1 |
| UBP | PtrUBP8.1 | Potri.016G067400 | 1227 | 408 | 4.88 | 46047.34 | nucleus | 16 |
| UBP | PtrUBP8.2 | Potri.006G201100 | 2829 | 942 | 5.01 | 107345.3 | cytosol | 6 |
| UBP | PtrUBP8.3 | Potri.001G214800 | 2655 | 884 | 5.40 | 99506.22 | nucleus | 1 |
| UBP | PtrUBP9 | Potri.001G449000 | 2817 | 938 | 5.19 | 105047.4 | nucleus | 1 |
| UBP | PtrUBP10 | Potri.011G152500 | 2829 | 942 | 5.44 | 105347.0 | nucleus | 11 |
| UBP | PtrUBP12.1 | Potri.006G198300 | 3348 | 1115 | 5.58 | 131373.8 | nucleus | 6 |
| UBP | PtrUBP12.2 | Potri.016G064100 | 3348 | 1115 | 5.54 | 131067.3 | cytosol | 16 |
| UBP | PtrUBP13.1 | Potri.008G012600 | 3516 | 1171 | 5.72 | 136580.5 | mitochondria | 8 |
| UBP | PtrUBP13.2 | Potri.010G245100 | 3513 | 1116 | 5.68 | 130749.7 | nucleus, cytoplasm | 10 |
| UBP | PtrUBP14.1 | Potri.011G125800 | 2418 | 805 | 5.00 | 89702.12 | mitochondria | 11 |
| UBP | PtrUBP14.2 | Potri.001G408800 | 2424 | 807 | 4.93 | 89745.45 | chloroplast, nucleus | 1 |
| UBP | PtrUBP15.1 | Potri.001G378900 | 2814 | 937 | 8.32 | 104330.8 | nucleus | 1 |
| UBP | PtrUBP15.2 | Potri.011G095200 | 2496 | 831 | 7.61 | 92149.2 | nucleus | 11 |
| UBP | PtrUBP16 | Potri.002G104800 | 3426 | 1141 | 8.14 | 124114.5 | chloroplast, nucleus  nunucleus | 2 |
| UBP | PtrUBP17 | Potri.005G156900 | 3378 | 1125 | 6.51 | 121979.7 | vacuole | 5 |
| UBP | PtrUBP18.1 | Potri.018G009500 | 2181 | 726 | 5.33 | 79907.58 | nucleus | 18 |
| UBP | PtrUBP18.2 | Potri.018G011200 | 1470 | 489 | 6.75 | 54888.45 | endoplasmic reticulum | 18 |
| UBP | PtrUBP19 | Potri.006G270600 | 1902 | 633 | 5.31 | 70172.1 | chloroplast | 6 |
| UBP | PtrUBP20 | Potri.003G092400 | 2430 | 809 | 5.31 | 90719.56 | nucleus | 3 |
| UBP | PtrUBP21 | Potri.001G142000 | 2508 | 835 | 5.27 | 93581.6 | nucleus | 1 |
| UBP | PtrUBP22.1 | Potri.018G123200 | 1764 | 587 | 8.59 | 66096.89 | nucleus | 18 |
| UBP | PtrUBP22.2 | Potri.006G266100 | 1668 | 555 | 8.42 | 63801.75 | nucleus | 6 |
| UBP | PtrUBP22.3 | Potri.018G017000 | 1662 | 553 | 8.4 | 63397.46 | nucleus | 18 |
| UBP | PtrUBP23.1 | Potri.006G185200 | 2853 | 950 | 8.89 | 104806.4 | nucleus | 6 |
| UBP | PtrUBP23.2 | Potri.018G107500 | 2850 | 949 | 9.34 | 104936.0 | nucleus | 18 |
| UBP | PtrUBP24 | Potri.003G202800 | 1632 | 543 | 6.4 | 59434.23 | cytosol | 3 |
| UBP | PtrUBP25.1 | Potri.011G112800 | 1974 | 657 | 9.03 | 72492.98 | chloroplast | 11 |
| UBP | PtrUBP25.2 | Potri.001G394600 | 1926 | 641 | 8.88 | 70967.01 | chloroplast | 1 |
| UBP | PtrUBP26.1 | Potri.005G179500 | 3255 | 1084 | 6.11 | 121468.9 | mitochondria | 5 |
| UBP | PtrUBP26.2 | Potri.002G081600 | 3258 | 1085 | 6.22 | 121337.2 | nucleus | 2 |
| UBP | PtrUBP27 | Potri.007G077100 | 1620 | 539 | 5.96 | 60895.59 | extracellular | 7 |
| UBP | PtrUBP28 | Potri.006G010100 | 1674 | 557 | 5.83 | 64713.28 | cytosol | 6 |
| UBP | PtrUBP29 | Potri.016G014000 | 1638 | 545 | 5.58 | 63370.81 | cytosol | 16 |
| UBP | PtrUBP30 | Potri.015G066500 | 4722 | 1573 | 5.46 | 176909.02 | nucleus | 15 |
| UBP | PtrUBP31 | Potri.012G071900 | 4635 | 1544 | 5.52 | 174203.14 | nucleus | 12 |
| UBP | PtrUBP32 | Potri.017G089250 | 6303 | 2100 | 5.44 | 239341.43 | nucleus | 17 |
| UCH | PtrUCH1 | Potri.004G130400 | 1005 | 334 | 5.99 | 38389.2 | cytosol | 4 |
| UCH | PtrUCH2 | Potri.017G075200 | 1005 | 334 | 5.75 | 38348.07 | cytosol | 17 |
| UCH | PtrUCH3 | Potri.003G081000 | 699 | 232 | 4.53 | 25324.61 | cytosol | 3 |
| OTU | PtrOTU1.1 | Potri.003G162600 | 882 | 293 | 4.64 | 33028.88 | nucleus | 3 |
| OTU | PtrOTU1.2 | Potri.001G067400 | 858 | 285 | 4.74 | 32270.4 | nucleus | 1 |
| OTU | PtrOTU2.1 | Potri.011G139500 | 726 | 241 | 5.29 | 26983.53 | cytosol | 11 |
| OTU | PtrOTU2.2 | Potri.001G430200 | 444 | 147 | 4.67 | 16707.84 | cytosol | 1 |
| OTU | PtrOTU2.3 | Potri.001G435500 | 627 | 208 | 4.9 | 23513.36 | cytosol | 1 |
| OTU | PtrOTU3 | Potri.016G110400 | 681 | 226 | 8.97 | 25616.25 | nucleus | 16 |
| OTU | PtrOTU4.1 | Potri.016G050900 | 1029 | 342 | 8.88 | 37955.08 | chloroplast | 16 |
| OTU | PtrOTU4.2 | Potri.006G057400 | 1212 | 303 | 9.63 | 34235.05 | chloroplast | 6 |
| OTU | PtrOTU4.3 | Potri.008G177400 | 492 | 163 | 8.85 | 18732.46 | cytosol | 8 |
| OTU | PtrOTU4.4 | Potri.010G234300 | 957 | 318 | 5.76 | 35963.03 | chloroplast | 10 |
| OTU | PtrOTU4.5 | Potri.008G026100 | 981 | 326 | 6.05 | 36874.26 | chloroplast | 8 |
| OTU | PtrOTU5 | Potri.014G134100 | 981 | 326 | 5.59 | 36659.82 | nucleus | 14 |
| OTU | PtrOTLD1.1 | Potri.009G160100 | 1605 | 534 | 5.05 | 58738.69 | chloroplast | 9 |
| OTU | PtrOTLD1.2 | Potri.004G196800 | 1608 | 535 | 4.94 | 58529.95 | nucleus | 4 |
| OTU | PtrOTU7 | Potri.005G140500 | 1155 | 384 | 6.37 | 42740.81 | nucleus | 5 |
| OTU | PtrOTU9.1 | Potri.008G036900 | 555 | 184 | 5.37 | 21158.97 | nucleus | 8 |
| OTU | PtrOTU9.2 | Potri.008G036700 | 1086 | 361 | 4.76 | 41450.21 | nucleus | 8 |
| OTU | PtrOTU9.3 | Potri.010G225400 | 1083 | 360 | 4.76 | 41021.39 | nucleus | 10 |
| OTU | PtrOTU10.1 | Potri.016G094700 | 1017 | 338 | 4.75 | 38991.68 | nucleus | 16 |
| OTU | PtrOTU10.2 | Potri.006G125900 | 1062 | 353 | 4.71 | 40686.63 | nucleus | 6 |
| OTU | PtrOTU11.1 | Potri.016G019700 | 693 | 230 | 7.13 | 26414.60 | nucleus | 16 |
| OTU | PtrOTU11.2 | Potri.006G021700 | 681 | 226 | 7.13 | 25798.05 | nucleus | 6 |
| OTU | PtrOTU12 | Potri.014G140200 | 681 | 226 | 9.00 | 26085.80 | nucleus | 14 |
| MJD | PtrMJD1 | Potri.001G249400 | 549 | 182 | 7.02 | 20971.02 | cytosol | 1 |
| MJD | PtrMJD2 | Potri.006G095700 | 696 | 231 | 4.51 | 25897.92 | nucleus | 6 |
| MJD | PtrMJD3 | Potri.009G043400 | 339 | 112 | 9.77 | 12771.85 | cytosol | 9 |
| MJD | PtrMJD4 | Potri.016G110300 | 843 | 280 | 4.45 | 31210.47 | nucleus | 16 |
| JAMM | PtrBRCC36AA | Potri.001G172800 | 1305 | 434 | 5.82 | 47367.14 | nucleus | 1 |
| JAMM | PtrBRCC36B | Potri.002G233500 | 1023 | 340 | 4.83 | 38881.34 | chloroplast | 2 |
| JAMM | PtrBRCC36C | Potri.014G147100 | 1026 | 341 | 4.73 | 39215.61 | chloroplast | 14 |
| JAMM | PtrBRCC36D | Potri.010G192000 | 864 | 287 | 4.88 | 31535.87 | cytosol | 10 |
| JAMM | PtrBRCC36E | Potri.010G192200 | 933 | 310 | 5.85 | 34822.22 | cytosol | 10 |
| JAMM | PtrBRCC36F | Potri.008G065300 | 933 | 310 | 5.96 | 34836.25 | chloroplast | 8 |
| JAMM | PtrBRCC36G | Potri.008G065200 | 933 | 310 | 5.76 | 34990.33 | cytosol | 8 |
| JAMM | PtrRPN11.1 | Potri.002G127900 | 939 | 312 | 6.31 | 34822.05 | cytosol | 2 |
| JAMM | PtrRPN11.2 | Potri.014G032900 | 939 | 312 | 6.31 | 34821.07 | cytosol | 14 |
| JAMM | PtrAMSH1 | Potri.015G045800 | 1545 | 514 | 7.21 | 58188.23 | nucleus | 15 |
| JAMM | PtrAMSH2 | Potri.010G041200 | 609 | 202 | 4.60 | 22556.11 | nucleus | 10 |
| JAMM | PtrAMSH3 | Potri.010G141100 | 1512 | 503 | 6.00 | 56958.20 | nucleus | 10 |
| JAMM | PtrCSN5A | Potri.018G006100 | 1101 | 366 | 5.17 | 40888.00 | nucleus | 18 |
| JAMM | PtrCSN5B | Potri.006G275100 | 1101 | 366 | 5.04 | 40878.89 | nucleus | 6 |
