# Supplementary material for: The DUB family in Populus: identification, characterization, evolution and expression patterns
Source: BMC Genomics. 2021 Jul 15;22:541. doi: 10.1186/s12864-021-07844-3 (PMC8281628; doi:10.1186/s12864-021-07844-3)
Supplement: Supplementary file 2 — Additional file 2 Table S2. Detailed information of on the Arabidopsis DUB family. [file 12864_2021_7844_MOESM2_ESM.docx]

Table S2. Detailed information of on the *Arabidopsis* DUB family.

| Species and family | | Gene ID | Length of amino acid | PI | Mw |
| --- | --- | --- | --- | --- | --- |
| *UBP* | *AtUBP1* | AT2G32780 | 1083 | 4.84 | 120770.70 |
|  | *AtUBP2* | AT1G04860 | 961 | 5.14 | 105152.35 |
|  | *AtUBP3* | AT4G39910 | 371 | 6.08 | 42447.14 |
|  | *AtUBP4* | AT2G22310 | 328 | 6.17 | 37863.14 |
|  | *AtUBP5* | AT2G40930 | 924 | 5.52 | 103874.91 |
|  | *AtUBP6* | AT1G51710 | 482 | 5.82 | 53695.90 |
|  | *AtUBP7* | AT3G21280 | 532 | 5.97 | 59832.16 |
|  | *AtUBP8* | AT5G22030 | 871 | 5.20 | 98170.96 |
|  | *AtUBP9* | AT4G10570 | 923 | 5.12 | 103698.56 |
|  | *AtUBP10* | AT4G10590 | 910 | 5.06 | 102649.40 |
|  | *AtUBP11* | AT1G32850 | 892 | 5.48 | 101175.16 |
|  | *AtUBP12* | AT5G06600 | 1116 | 5.51 | 130606.95 |
|  | *AtUBP13* | AT3G11910 | 1115 | 5.38 | 130648.82 |
|  | *AtUBP14* | AT3G20630 | 797 | 5.05 | 88373.84 |
|  | *AtUBP15* | AT1G17110 | 924 | 8.01 | 103676.50 |
|  | *AtUBP16* | AT4G24560 | 1008 | 6.73 | 110600.32 |
|  | *AtUBP17* | AT5G65450 | 731 | 8.60 | 81998.98 |
|  | *AtUBP18* | AT4G31670 | 631 | 4.97 | 70752.59 |
|  | *AtUBP19* | AT2G24640 | 672 | 4.98 | 75719.55 |
|  | *AtUBP20* | AT4G17895 | 695 | 4.45 | 78632.91 |
|  | *AtUBP21* | AT5G46740 | 732 | 5.97 | 82417.13 |
|  | *AtUBP22* | AT5G10790 | 557 | 8.03 | 63517.52 |
|  | *AtUBP23* | AT5G57990 | 859 | 9.31 | 94903.11 |
|  | *AtUBP24* | AT4G30890 | 551 | 7.70 | 60440.05 |
|  | *AtUBP25* | AT3G14400 | 661 | 9.21 | 73293.14 |
|  | *AtUBP26* | AT3G49600 | 1046 | 5.73 | 117566.12 |
|  | *AtUBP27* | AT4G39370 | 505 | 6.29 | 57035.26 |
| *UCH* | *AtUCH1* | AT5G16310 | 334 | 5.08 | 38539.05 |
|  | *AtUCH2* | AT1G65650 | 330 | 5.03 | 37498.76 |
|  | *AtUCH3* | AT4G17510 | 234 | 4.91 | 25712.24 |
| *OTU* | *AtOTU1* | AT1G28120 | 306 | 4.55 | 34434.46 |
|  | *AtOTU2* | AT1G50670 | 208 | 4.98 | 23425.45 |
|  | *AtOTU3* | AT2G38025 | 234 | 9.47 | 26272.94 |
|  | *AtOTU4* | AT3G57810 | 317 | 8.98 | 35773.65 |
|  | *AtOTU5* | AT3G62940 | 332 | 6.86 | 37361.73 |
|  | *AtOTLD1* | AT2G27350 | 506 | 5.00 | 55275.55 |
|  | *AtOTU7* | AT5G67170 | 375 | 6.13 | 41533.54 |
|  | *AtOTU8* | AT2G39320 | 189 | 9.49 | 22397.67 |
|  | *AtOTU9* | AT5G04250 | 345 | 4.90 | 39231.44 |
|  | *AtOTU10* | AT5G03330 | 356 | 4.81 | 41689.31 |
|  | *AtOTU11* | AT3G22260 | 245 | 8.97 | 28227.66 |
|  | *AtOTU12* | AT3G02070 | 219 | 9.13 | 25571.32 |
| *MJD* | *AtMJD1* | AT1G07300 | 128 | 10.86 | 13890.02 |
|  | *AtMJD2* | AT2G29640 | 360 | 9.42 | 40489.26 |
|  | *AtMJD3* | AT3G54130 | 280 | 4.64 | 30692.06 |
| *JAMM* | *AtAMSH1* | AT1G48790 | 507 | 6.26 | 57383.25 |
|  | *AtAMSH2* | AT1G10600 | 223 | 5.07 | 24938.94 |
|  | *AtAMSH3* | AT4G16144 | 422 | 6.17 | 47355.02 |
|  | *AtBRCC36A* | AT1G80210 | 406 | 5.81 | 44483.65 |
|  | *AtBRCC36B* | AT3G06820 | 405 | 5.91 | 44411.81 |
|  | *AtCSN5A* | AT1G22920 | 357 | 5.11 | 39602.32 |
|  | *AtCSN5B* | AT1G71230 | 358 | 5.05 | 40262.99 |
|  | *AtRPN11* | AT5G23540 | 308 | 6.31 | 34353.36 |
