# Supplementary material for: The DUB family in Populus: identification, characterization, evolution and expression patterns
Source: BMC Genomics. 2021 Jul 15;22:541. doi: 10.1186/s12864-021-07844-3 (PMC8281628; doi:10.1186/s12864-021-07844-3)
Supplement: Supplementary file 3 — Additional file 3 Table S3. Detailed information on the MEME motif sequences about DUB proteins in Populus trichocarpa. [file 12864_2021_7844_MOESM3_ESM.docx]

Table S3. Detailed information on the MEME motif sequences about DUB proteins in *Populus trichocarpa*.

| **Motif** | **Width** | **Best possible match sequences** |
| --- | --- | --- |
| 1 | 29 | AGLQNLGNTCFMNSVLQCLTHTPPLRNYL |
| 2 | 29 | KCKEKVDARKKLSJWRAPNVLVJHLKRFE |
| 3 | 29 | FGGQLRSTVVCPNCNKKSTRYEPFMDLSL |
| 4 | 16 | QEDAHEFLRFLLDGLH |
| 5 | 15 | HGGGLHSGHYVAYVK |
| 6 | 21 | VSPVSEEEVLSSAAYMLFYSR |
| 7 | 29 | DVGGAVSLEDCLDQFTKPERLEGDNKYYC |
| 8 | 50 | SYVCANCFSPGTTRCSRCKSVRYCSGKCQIIHWRQGHKEECKDFKTTSSQ |
| 9 | 29 | LWSPGRTPIAPRQFKMKLAKFAPQFSGYN |
| 10 | 50 | MTVTVYDPDGSELPYPGTVTVPKSGKPRDJJAALSIACSLKNDEEJELAE |
| 11 | 29 | YCKKMRKPGEWGGHVELQAAADVYGRKIC |
| 12 | 21 | GLEERKVPGDGNCLFRALADQ |
| 13 | 50 | FKDTCYIEILPKSQKPKRVIFLSFWAEVHYNSJYEZGDVPTEEPKKKKWW |
| 14 | 29 | ELHKLVREQVVDZLKKRREEYEGFVPMDY |
| 15 | 29 | HNYHENMFSMLKQVNRKENVVGWYHSHPG |
| 16 | 41 | VGKRJSQLVPIPHVPRINGEIPSEDEATSDHZRLLERLKVY |
| 17 | 33 | CFLSGIDIHTQMSYQAMLPRAVAIVMDPTRSVS |
| 18 | 41 | PPTSETPBPLRQVQISALALLKMIKHARAGGPIEVMGLMLG |
| 19 | 50 | IQTMRCDLYGQERTYSERAMLIYDGLHYDALAMSPFEGAPEEFDQTIFAV |
| 20 | 18 | ENPIRVLYHGYGHYDILV |
| 21 | 50 | NKQYYSJDITYRKNELDCKLLDLLWKKKWVDGLSLSPFDGNGDYNEGTIQ |
| 22 | 50 | RENDLEIHGLFNDYVPNPVLVIIDVQPEELGIPTKAYYAVEEVKENATQK |
| 23 | 41 | NYPDLVIASNVPLBHEIARTLQEEFSRLANLNTEDJLKAFA |
| 24 | 28 | EDKGKEHLLRDVKDTTIASDPEEYTGKL |
| 25 | 41 | IVDNYNRVAKDTRKRVVGVLLGSSFKGTVDVTNSYAVPFEE |
| 26 | 47 | KTNDMMLVIYLSSLIRSVIALHNLINNKMLNKEHEKAEDSKPVAVPP |
| 27 | 29 | HLHYHWICYRKVDGVWYELDGLKEGPISL |
| 28 | 50 | IWHVIQPADWRRRRERRSVRGEGSWNVAWDGRPARWLHRPDSAWLLFGVC |
| 29 | 21 | VYMRDRTSGGLINIAEYGQEY |
| 30 | 50 | QJLESRDGPLVDPQKKKDEESRKAKQSRGYVDFTFEDFHGLFLZQJDDIL |
| 31 | 33 | EPHNNSFGGDFDIQVLQKALEEWDKQVIAHDSP |
| 32 | 50 | VAEHAQIDPDLENAFICHLHDHWFCIRKVNGEWYNFDSLYAAPQHLSKFY |
| 33 | 50 | NGGMLYHEVQEAKLCAVHCVNTVLQGPFFSEFDLAALASDLDSKERQMMQ |
| 34 | 50 | GWSIFLVRGNFPKEFPIESSEAPNGYGQWLSPEDAERIIKSCNNTZSPQR |
