# Supplementary figures and images for: The DUB family in Populus: identification, characterization, evolution and expression patterns
Source: BMC Genomics. 2021 Jul 15;22:541. doi: 10.1186/s12864-021-07844-3 (PMC8281628; doi:10.1186/s12864-021-07844-3)

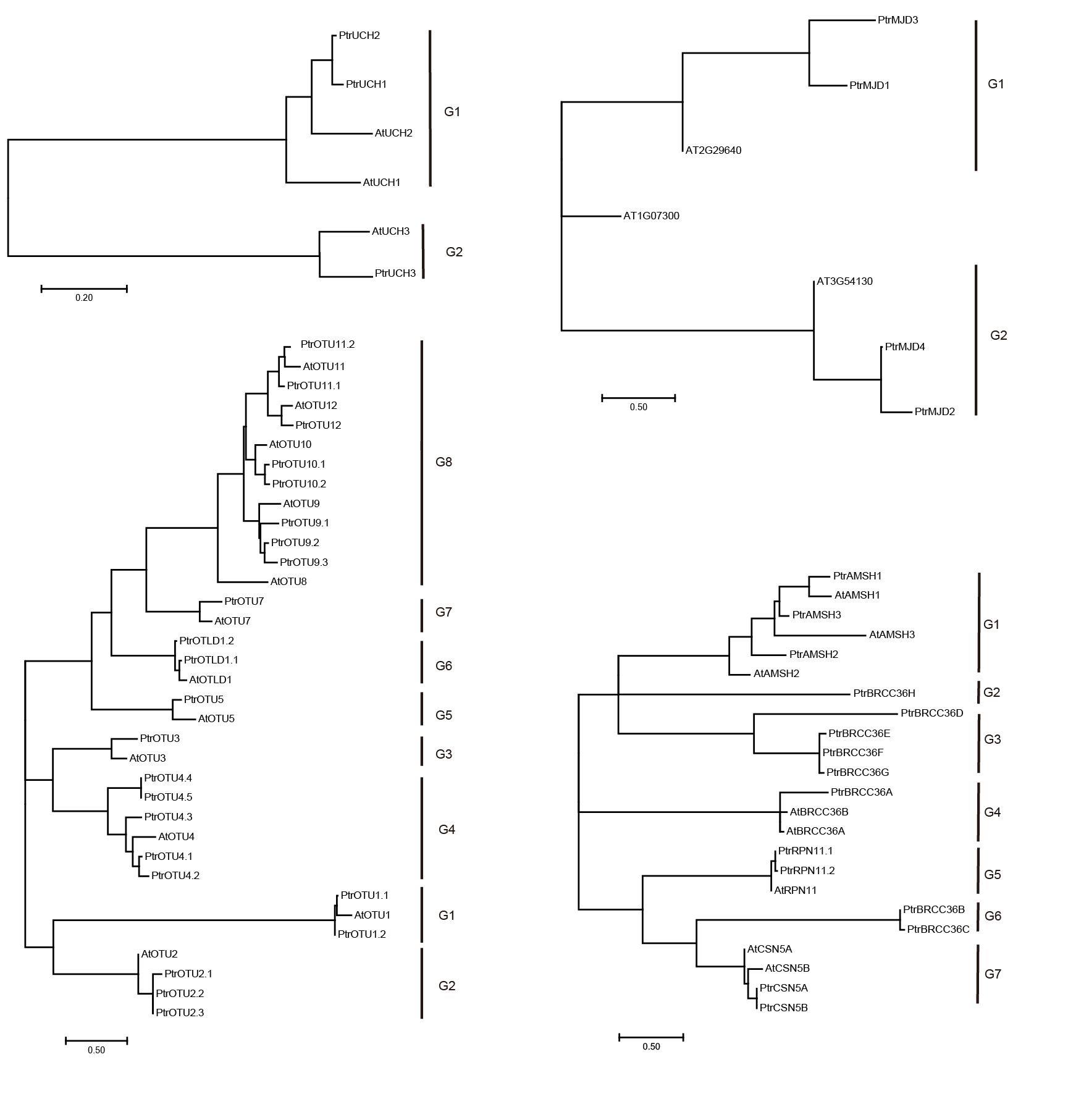

Supplement: Supplementary file 7 — Additional file 7 Fig. S1 Evolutionary relationships among Poplus trichocarpa and Arabidopsis thaliana DUB subfamily members. [file 12864_2021_7844_MOESM7_ESM.jpg]
